# The coming of age of the pediatric EBMT criteria

**DOI:** 10.1038/s41409-020-01141-5

**Published:** 2020-11-21

**Authors:** Selim Corbacioglu

**Affiliations:** Department of Pediatric Hematology, Oncology and Stem Cell Transplantation, University of Regensburg, Franz-Josef-Strauß-Allee 11, 93053 Regensburg, Germany

**Keywords:** Haematological diseases, Stem-cell research

Sinusoidal obstruction syndrome also known as veno-occlusive disease (SOS/VOD) is a life-threatening complication after hematopoietic stem cell transplantation (HSCT). The classical clinical presentation of SOS/VOD consists of hyperbilirubinemia, hepatomegaly, with right upper quadrant pain, ascites and weight gain as the consequence of this primary endothelial injury. Eighty percent of SOS/VOD develops before day 30 after HSCT, but it can be diagnosed later. Due to the lack of sensitive and specific biological markers or imaging tools, SOS/VOD remains a clinical diagnosis.

Seattle criteria and Baltimore criteria to diagnose SOS/VOD have been used unanimously for adults and children for the last decades, with minor adjustments. Several shortcomings of these criteria were identified over the last decades, such as the obligatory hyperbilirubinemia (>2 mg/dl) of the Baltimore criteria, the time limit of 20 and 21 days respectively, the arbitrary weight gain of 2 and 5% that were increasingly recognized as obsolete, in particular in the pediatric patient population.

The significant differences between adults and children in many aspects such as incidence, genetic predisposition, clinical presentation, prevention and treatment, as well as outcome were acknowledged recently by the European Society for Blood and Marrow Transplantation (EBMT). Consecutively, separate diagnostic criteria for adults [1] and children [2] were proposed by the EBMT.

The pediatric EBMT (pEBMT) criteria aimed predominantly at clinical aspects that have not been considered so far. Anicteric VOD/SOS is observed in 30% of children but also increasingly recognized in adult patients before day +21 post-HSCT [3]. Hepatomegaly and ascites are frequent pre-transplant conditions in hemophagocytic lymphohistiocytosis and patients with hemoglobinopathies so that precise pre-HSCT baseline measures are mandatory. A frequently observed and very sensitive clinical marker is transfusion refractory thrombocytopenia (TRT), already described by McDonalds et al. [4], but that was neither considered as a diagnostic nor as severity marker. TRT is now increasingly recognized as a sensitive predictor of VOD/SOS and was therefore integrated in the pEBMT criteria.

The major intention of the pEBMT criteria was to trigger early/preemptive intervention with defibrotide (DF). DF demonstrated efficacy for the treatment and prevention of SOS/VOD and received European Medicines Agency authorization for the treatment of SOS/VOD. The importance of timing of intervention with DF was shown in several trials, demonstrating that early intervention with DF is associated with a superior outcome [5–7].

This preemptive approach of the pEBMT criteria inevitably provokes an adjustment of the incidence of VOD/SOS but is intended to improve outcome with regard to morbidity, severity of diseases, hospitalization and mortality. Highly variable incidences of SOS/VOD depending on the criteria used for diagnosis are well known from Seattle and Baltimore criteria with variations of up to 400% [8, 9] but are comprehensible with the prevalence of an anicteric presentation.

Until recently the proposed criteria were lacking evidence. Recently, Embaby et al. [10] demonstrated that refractory thrombocytopenia is a sensitivity early marker and Szmit et al. [11] showed in a prospective comparison of the Seattle and pEBMT criteria a higher DF response rate, a significantly improved overall survival and a lower transplant related morbidity.

In this Journal, Ragoonanan et al. [12] compared retrospectively the variations of key parameters in the diagnosis of VOD/SOS using the Baltimore, Seattle and pEBMT criteria in 226 patients treated at a large academic institution in the United States who underwent HSCT between July 2009 and 2019. Albeit the incidence of VOD/SOS was slightly higher (15,9%) using the pEBMT diagnostic criteria compared with the modified Seattle (12.3%), and Baltimore (6.6%) criteria, respectively, the time to diagnosis of VOD/SOS was by 2.5–3 days earlier. Refractory thrombocytopenia was present in 75% of patients at diagnosis. The sensitivity of this criterion was underlined by the fact that approximately 61% of patients with VOD/SOS were anicteric at diagnosis but the majority (94.4%) developed hyperbilirubinemia over a median time of 4 (1–57) days, explaining the significant discrepancy to the published incidences of anicteric VOD/SOS in children. The authors conclude that application of pEBMT criteria may have resulted in earlier indication for definitive treatment by 3 days and that in their hands the pEBMT criteria were sensitive and highly specific.

Taken together, the recently accumulated, albeit retrospective, data on the sensitivity and specificity of the pEBMT are encouraging but need prospective confirmatory evidence since baseline ultrasound was rarely standard of care in the past and TRT is difficult to assess from patients records properly. With regard to anicteric VOD/SOS and TRT being also prevalent in adults, an update of the diagnostic criteria might be worth a consideration.
